# Simulation of Autonomous Underwater Vehicles (AUVs) Swarm Diffusion

**DOI:** 10.3390/s20174950

**Published:** 2020-09-01

**Authors:** Enrico Petritoli, Marco Cagnetti, Fabio Leccese

**Affiliations:** Science Department, Università degli Studi “Roma Tre”, Via della Vasca Navale n. 84, 00146 Rome, Italy; e_petritoli@libero.it (E.P.); ing.marco.cagnetti@gmail.com (M.C.)

**Keywords:** autonomous underwater vehicles, AUV, swarm, diffusion

## Abstract

The paper shows the simulation of the behavior of a swarm of underwater drones (AUV) diffused in a closed section of the sea and inserted from a single starting point: Based on a few essential rules, we will see how their behavior evolves and how they manage to spread throughout the area assigned to them. In the first part of this work, after defining the design of the vehicle, we introduce our vision of the swarm, its problems, and its strengths. Later, we show how to spread a series of underwater drones with “diffused intelligence” (swarm) and its microscopic diffusion model. In the last part, we present the simulation that supports our approach to the swarm.

## 1. Introduction

This paper is part of several preliminary studies by the Underwater Drones Group (UDG) of the Science Department of the Università degli Studi “Roma Tre,” which is developing several advanced autonomous underwater vehicles (AUVs) for the exploration of deep seas. The final aim of the general project is to create modular task platforms for underwater scientific research that can accommodate a wide range of different payloads optimized for the most missions.

In this work, we have proposed a new type of AUV swarm and simulated its use in the Channel of Piombino out of the coast of Tuscany (Italy). The architecture of the vehicle, designed for shallow and white waters, will first be illustrated. Then, the swarm architecture and its advantages from the point of view of reliability and operational use will be developed: After that, we will show how to spread a series of underwater drones with “diffused intelligence” (swarm) and its microscopic diffusion model in a closed section of the sea when they are inserted from a single starting point. At the end, we are going to present the simulation that confirms that our approach to the swarm is capable of spreading homogeneously over a specific closed area.

State-of-the-Art and New Approach

Sometimes, science tries to copy nature; in fact, when drones (intended as autonomous vehicles) have enjoyed a technology that makes them small, economical, and intelligent enough, their use in swarms was a natural step. 

From the point of view of the philosophy of use, the first step was to imitate and borrow the behavior of flocks of birds in the sky or schools of fish in the sea. All this has led to a drone development philosophy in which the single individual (vehicle) collaborates with others in the name of a “common good” and all the components of the swarm combine to accomplish a mission [1]. This approach brings precise logical and technological constraints: First of all, a close collaboration between the proximal vehicles, which, in turn, entails a considerable flow of information that must be exchanged. Secondly, each vehicle has a particular task: It is certainly replaceable by the others but, at the moment, it covers that role whereby its “social position” must be transmitted to the other members, and this imposes another penalty in the aggravation of the weight of communications. Due to the great flexibility, it is possible to divide, to satisfy small momentary tasks, the swarm into smaller patrols: In this case as well, a heavy price is paid in terms of communication flow and the general arrangement of the various groups [2,3,4,5]. 

For some time, however, the philosophy of the approach to the problem has been changing: Now, the group of drones is seen as composed of more autonomous elements, with much looser mutual constraints: The personal initiative of the individual vehicle becomes, from a philosophical-point-of-view implementation, much more important than “choral work”: Despite this radical change, it is noted that the swarm is able, in any case, to carry out the mission assigned to it and, often, in a faster and more efficient way [6,7,8,9,10].

All this is present in the paradigm of our swarm: The individual components can be considered “selfish” as they do not take into account their fellowmen unless they get too close: In that case, a repulsion occurs. On the other hand, the single vehicle, if it is unable to carry out a mission, “commits suicide” by isolating itself and returning to base. In this paper, the authors propose the study of a similar paradigm of swarm behavior in an underwater environment that, at the moment, has not yet been investigated. 

## 2. Materials and Methods

### 2.1. The Simulation Environment

The Mediterranean Sea is a peculiar environment: The bathymetry values of the Sea have a very wide range of variations, e.g., the continental shelf (like the Adriatic Sea and the Tunisian platform) has a depth below 100 m; in other areas, like the Tyrrhenian Sea, the Ionian Sea, and some areas of the Near East Sea, depths reach 4000–5000 m. The difference between the Mediterranean and the open oceans lies in the great variety of depth and variation-of-use scenarios, within a narrow area. From an oceanographic point of view, the Mediterranean Sea is a “concentration basin”: The losses of water due to evaporation exceed the water entrances coming from rivers and rains. 

To compensate the increase in density and the decrease in the average sea level with respect to the Atlantic Ocean, a large number of underwater currents have been activated not only horizontally, but also vertically, causing, seasonally, a strong mixing of the salt layers. An area between the port of Piombino and Elba Island was chosen, a square area of 36 km^2^ (6 km × 6 km). It is also an area densely traversed by both transport and cruise ships; therefore, it is necessary to often monitor the health of the sea, discover and trace the presence of oil leaks or other contaminants, and monitor recreational maritime traffic. 

A simulation of a swarm of drones has been programmed not to leave the area except for its own failure. 

### 2.2. The Vehicle

#### 2.2.1. General Description

**Figure 1:**
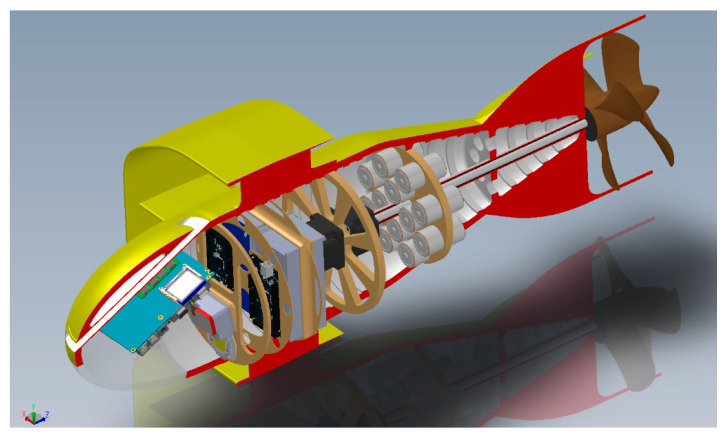
Prospective cross-section of the autonomous underwater vehicles (AUV) Albacore: The interior sections and supports are visible.

The vehicle is a cylindrical AUV, with an annular wing and propelled by a double electric motor. Let us look at its detailed description (see Figure 1 below).

In the front bay, there is a radome with a “nostril” that houses the sensors kit: The data collected are managed by a computer. The nostril is inclined at 20° so that its flow is the least disturbed possible and its discharge flow does not create turbulence or disturbance to the flow of the elliptical wing. Below, there is the corresponding window of a digital camera (GoPro class) for visual inspection and automatic recognition of objects at depths, which, although modest, could be lacking in sufficient light; on the first bulkhead, a 10^6^ candle, flat LED has been mounted [11,12,13,14,15,16]. Lighting for the camera is important in the case of operation in the low waters of the ports, notoriously turbid or, in co-current sources, coming from silty river mouths [17]. The general characteristics and performances (estimated) of our underwater vehicle are shown in Table 1.

The tasks of a single AUV are various: From the autonomous surveillance of the networks (thanks to the recognition system from self-learning - neural network) to the sentinel function for oil spills or biological contaminations. Furthermore, it could be able to identify fish schools or the presence of boats or great ships. The vehicle also has a peculiar use: In the case of biochemical contamination, it is able to recognize it and, partially emerging, take a picture above the free surface of the water of all the ships present at that time in the area [18].

#### 2.2.2. Short-Range Communications

Short-distance communication between the vehicles is based on an optical laser communication system. Although it is not the primary focus of this paper, it is useful to spend a few words to describe the general architecture. Due to the low dynamics (speed) of the drones, when they approach, it is possible that they establish UOWC (underwater optical wireless communication) contact in which they exchange different data, which will then serve to diverge and avoid collision. A 520 nm laser diode (LD) is a simply modulated NRZ-OOK (Non-Return-to-Zero On–Off Keying): According to [19], we have a data rate of 500 Mbps with a BER (bit error rate) of 2.5 × 10^−3^ over 100 m well below the forward-error correction of 3.8 × 10^−3^ with 7% overhead.

**Figure 2:**
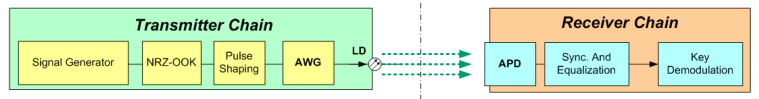
Short Range Optical Communication System: transmitter/receiver chain.

From a functional point of view, the Rx-Tx chain is described in Figure 2: It starts from the signal generator that, through the “NRZ-OOK” unit, passes to pulse shaping and then to the AWG (Arbitrary Wave Generator), which carries the signal to the laser diode: Downstream, we will find the focusing optical group (absent in the figure for clarity). The signal, revealed by the APD (avalanche photodiode), once synchronized and equalized, is sent to the key demodulation and, from there, to the navigation system of the drone. 

**Figure 3:**
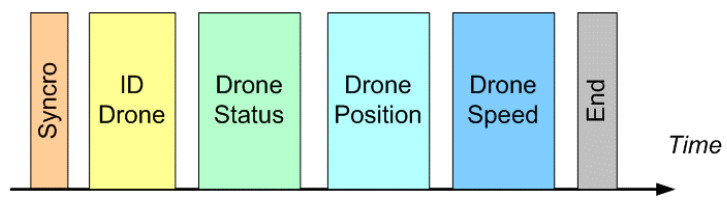
Short Range Optical Communication System: signal structure.

The signal exchanged is very essential (as seen from Figure 3 below): It contains the identification code of the drone, information regarding its electromechanical “health” and, last but not least, position and speed, supported by its IMU (Inertial Measurement Unit). At this point, the two drones have all the information necessary to change direction according to the protocols indicated.

### 2.3. The Swarm Architecture

We designed a “swarm intelligence” approach for our system: The behavior of the single drone is similar to social animals that solve problems or perform a task thanks to interaction with the other members of the group. In our case, this takes on the appearance and the collective strategy of a swarm of insects.

We avoided concentrating all the necessary intelligence on a single vehicle, otherwise, it would have become too big and expensive: Instead, we spread it over a larger number of machines whose relatively modest cost makes it an expendable item if necessary. For this purpose, we imagine using it in areas with strong contamination, or near intense flames or highly corrosive substances.

The strengths of the “swarm paradigm” are basically six:

1. the swarm is composed of a large number of vehicles (depending on the desired density);
2. all swarm vehicles are the same and interchangeable between them;
3. each swarm vehicle is placed independently in its own position: Its fix point (starting point for performing a task) is not predetermined;
4. the single vehicle is “stupid” if taken individually and is simple to operate;
5. the single vehicle has its own sensors and is programmed to communicate with a reduced number of “neighbors”: Communication with the central control takes place only in an emergency or in the event of serious malfunctions;
6. each drone goes offline autonomously when it self-assesses that it is unable to carry out the mission, being equipped with internal self-purging routines.

From the operative point of view instead, the most important advantages of a drone swarm are:

- **Reliability**: The overall reliability of the system is very high.
- **Scalability**: Every component of the swarm can easily engage in a task or disengage without affecting the efficiency of the swarm or the performance of a task.
- **Parallelism**: The swarm can perform more than one task simultaneously and also different and diversified tasks.
- **Economy**: The single UAV has an extremely reduced cost for design, for its small size and for low complexity.

One of the main strengths of the “swarm paradigm” is precisely the fact that the need to program every single drone in detail is lacking: Each of these instead constitutes an entity that is granted great decision-making autonomy on a small scale. We, therefore, have two operational levels, one “tactical” and the other “strategic”: We decide the strategy on a higher scale level, that is, we decide what is accomplished with the fulfillment of the final mission, which can be the monitoring of a stretch of coast or the analysis of substances dispersed in marine water. The drone, on the other hand, operates on a small scale, precisely on a tactical level: It is programmed with an extremely simple philosophy and invited to “get away with it” without having to bring your decisions back to a higher level; moreover, it will decide in complete autonomy if its state of “health” allows it to carry out a mission or not [19].

### 2.4. Reliability of the Swarm

We decided to carry out the mission using a swarm of drones all the same as each other, whose advantages have already been illustrated; now, we have to answer the question, “how many should we use”? The natural answer would be “as much as possible” but, for the cost and economy of scale, this solution is not applicable. We decided to base the approach on the final decision on the overall reliability of the “swarm system” or, given the reliability of the single drone, let us see what is the probability that the swarm will complete the mission: Having set a minimum limit, we can determine the overall number.

#### 2.4.1. General Reliability

In this section, we examine the swarm from a reliability point of view, considering it as an entity that performs a series of tasks and is composed by the same members.

The general formula used to calculate the reliability of a system of *N* items (or drones) is:

(1) $$R_{sys}\left(t\right)=\;1-{\left(1-e^{-\lambda t}\right)}^{N}$$

where:

*λ* is the failure rate of the single item (drone). 

*t* is the mission time.

Developing with Newton’s binomial:

(2) $$R_{sys}\left(t\right)=\;1-\;\left(1-\left(\begin{array}{c} N\\ 1 \end{array}\right)e^{-\lambda t}+\left(\begin{array}{c} N\\ 2 \end{array}\right)e^{-2\lambda t}-\left(\begin{array}{c} N\\ 3 \end{array}\right)e^{-3\lambda t}+\ldots \right)$$

In explicit form:

(3) $$R_{sys}\left(t\right)=N\;e^{-\lambda t}-\frac{N\left(N-1\right)}{2!}e^{-2\lambda t}+\frac{N\left(N-1\right)\left(N-2\right)}{3!}e^{-3\lambda t}-\ldots$$

The definition of *MTBF* (Mean Time Between Failures) of the whole system is:

(4) $$MTBF_{sys}=\overset{\infty }{\underset{0}{\int }}R_{sys}\left(t\right)\;dt$$

Thus, the equation becomes:

(5) $$MTBF_{sys}=\overset{\infty }{\underset{0}{\int }}\left(N\;e^{-\lambda t}-\frac{N\left(N-1\right)}{2!}e^{-2\lambda t}+\frac{N\left(N-1\right)\left(N-2\right)}{3!}e^{-3\lambda t}-\ldots \right)dt$$

In explicit form:

(6) $$MTBF_{sys}={\left[-\frac{N}{\lambda }\;e^{-\lambda_{sys}t}\right]}_{0}^{\infty }-{\left[-\frac{N\left(N-1\right)}{2!}\frac{1}{2\lambda }\;e^{-2\lambda t}\right]}_{0}^{\infty }+{\left[-\frac{N\left(N-1\right)\left(N-2\right)}{3!}\frac{1}{3\lambda }\;e^{-3\lambda t}\right]}_{0}^{\infty }-\ldots$$

Thus:

(7) $$MTBF_{sys}=\frac{N}{\lambda }-\frac{N\left(N-1\right)}{2!}\frac{1}{2\lambda }\;+\frac{N\left(N-1\right)\left(N-2\right)}{3!}\frac{1}{3\lambda }-\ldots$$

The expression of reliability for a system with *n* components in parallel with sequential operation can then be simply obtained as the probability of having, at most, *N −* 1 failures (drone failed) in the interval [0, …, *t*] of:

(8) $$R_{sys}\left(t\right)=\frac{{\left(\lambda t\right)}^{0}}{0!}\cdot e^{-\lambda t}+\frac{{\left(\lambda t\right)}^{1}}{1!}\cdot e^{-\lambda t}+\ldots +\frac{{\left(\lambda t\right)}^{N-1}}{\left(N-1\right)!}\cdot e^{-\lambda t}$$

Then:

(9) $$R_{sys}\left(t\right)=e^{-\lambda t}\cdot \left(1+\lambda t+\frac{{\left(\lambda t\right)}^{2}}{2!}+\ldots +\frac{{\left(\lambda t\right)}^{N-1}}{\left(N-1\right)!}\right)$$

The upper limit of the second member is:

(10) $$\underset{N\to \infty }{\lim}\left(1+\lambda t+\frac{{\left(\lambda t\right)}^{2}}{2!}+\ldots +\frac{{\left(\lambda t\right)}^{N-1}}{\left(N-1\right)!}\right)=e^{\lambda t}$$

therefore, the reliability of the system, as the number of components increases, tends to 1.

Finally, regarding the value of the MTBF, we have:

(11) $$MTBF_{sys_{N}}=\int_{0}^{\infty }e^{-\lambda t}\cdot \left(1+\lambda t+\frac{{\left(\lambda t\right)}^{2}}{2!}+\ldots +\frac{{\left(\lambda t\right)}^{N-1}}{\left(N-1\right)!}\right)dt$$

Thus, the solution is:

(12) $$MTBF_{sys_{N}}={\left[-\frac{1}{\lambda }\cdot e^{-\lambda t}\cdot \overset{N-1}{\underset{k=0}{\sum }}\frac{{\left(\lambda t\right)}^{k}}{k!}\;\;\right]}_{0}^{\infty }\;\;\;-\int_{0}^{\infty }\left[-\frac{1}{\lambda }\cdot e^{-\lambda t}\cdot \left(\overset{N-1}{\underset{k=0}{\sum }}\frac{{\left(\lambda t\right)}^{k}}{k!}\cdot k\lambda \right)\right]dt$$

Thus:

(13) $$MTBF_{sys_{N}}=\frac{1}{\lambda }-\int_{0}^{\infty }\left[-\frac{1}{\lambda }\cdot e^{-\lambda t}\cdot \left(\overset{N-1}{\underset{k=0}{\sum }}\frac{{\left(\lambda t\right)}^{k}}{k!}\cdot k\lambda \right)\right]dt$$

Simplifying:

(14) $$MTBF_{sys_{N}}=\frac{1}{\lambda }+\int_{0}^{\infty }\left[e^{-\lambda t}\cdot \left(\overset{N-1}{\underset{k=0}{\sum }}\frac{{\left(\lambda t\right)}^{k-1}}{\left(k-1\right)!}\right)\right]dt$$

Then:

(15) $$MTBF_{sys_{N}}=\frac{1}{\lambda }+\int_{0}^{\infty }\left[e^{-\lambda t}\cdot \left(\frac{{\left(\lambda t\right)}^{-1}}{\left(-1\right)!}+\overset{N-1}{\underset{k=1}{\sum }}\frac{{\left(\lambda t\right)}^{k-1}}{\left(k-1\right)!}\right)\right]dt$$

Regroup the first member:

(16) $$MTBF_{sys_{N}}=\frac{1}{\lambda }+\int_{0}^{\infty }\left[e^{-\lambda t}\cdot \left(\overset{N-2}{\underset{k=0}{\sum }}\frac{{\left(\lambda t\right)}^{k}}{\left(k\right)!}\right)\right]dt$$

We then returned to the starting integral, which is, again, solved by parts, obtaining the value 1/*λ* and lowering the sum by degree. Ultimately, we have:

(17) $$MTBF_{sys_{N}}=\frac{1}{\lambda }+\ldots +\frac{1}{\lambda }+\int_{0}^{\infty }\left[e^{-\lambda t}\right]dt$$

Thus:

(18) $$MTBF_{sys_{N}}=\frac{N}{\lambda }$$

The reliability calculations seen previously are particularly significant for non-repairable components and systems, that is, for those cases in which the failure of the fault involves the replacement of the component or system: A classic case of non-repairable application is precisely a satellite system, as it is placed in an environment that prevents this type of operation. The analysis shows that a larger swarm is more capable (it may have a chance) of completing the mission.

#### 2.4.2. Reliability of K-out-of-*n* System

The simple reliability of the previous section is not practicable for us, because the loss of only one of the drones would lead to the loss of the whole mission and, therefore, for us, this is not acceptable. We have established that the minimum number of drones to complete the mission is 16 (k = 16): Assigning to each drone an area equal to 1.51 × 0.5 km, we obtain a perimeter equal to 6 km; in the worst case, the drone runs along the perimeter, at an average speed of 30 km/h, in just under 13 min. In our mission, the GPS checks take place at an average of one hour, so it has time to travel the perimeter four times, more than enough to detect what is required of it. Now, posing R = 0.9 as, according to the classical calculation, we will obtain insufficient reliability, we have to support it with a number of other drones so that it can complete the mission. The value 0.9 is a requirement that was placed on us by the Coast Guard when setting up the study on this system.

We now consider the main objective of this section: To obtain an expression for the reliability of the k-out-of-*n* system (drones):

(19) $$R_{k/n}=\overset{n}{\underset{i=k}{\sum }}\left(\begin{array}{c} n\\ i \end{array}\right)p^{i}{\left(1-p\right)}^{n-i}$$

where:

*n* = 25 is the number of items (drones). 

*k* = 16 is the minimum number of drones necessary to accomplish the mission

*p* = 1.651 × 10^–9^ is the probability of failure of the single drone at time *t* = 168 h

Thus, we have

$$R_{16/25}=0.981$$

Above the required reliability of $R_{required}=0$.9, the overall 1.651 × 10^–9^ value was deduced starting from the reliability value of the single drone whose calculation was performed some time ago by our working group, and it is not the subject of this paper [15].

**Self-purging Redundancy**

Our redundancy scheme is a self-purging redundancy, a scheme that uses a threshold voter based on fuzzy logic that “purges” the failed drones and declare it off-line.

**Figure 4:**
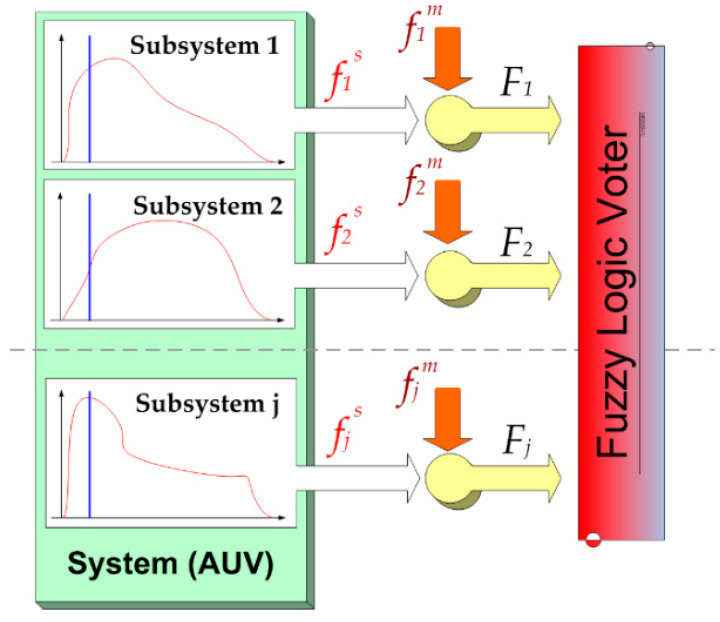
Fuzzy logic voter block diagram.

Each drone, at regular intervals, performs a BIT (Built-in Test) to check the operating status of all its subsystems. This is the first technical check that, if not passed, puts the drone off-line. Subsequent checks are necessary as these vehicles are designed for medium-long missions at sea and, therefore, the occurrence of a failure is high (see Figure 4).

Another BIT method lays the foundations on the prognostics of the subsystems: As digital systems also undergo analog degradation, today, the level of confidence with the electronics and on-board systems is extremely high, so we can have, in advance, the model of fin degradation of the single component. Therefore, from a reliability point of view, we can know what the “safety margin” of the subsystem in question is, as a function of the useful life that is decreasing over time. In other words, at time t, we can say that the subsystem “x” has a well-known percentage of useful life.

Not only that, but its degradation state $f_{i}^{s}$ is “weighed” with another parameter $f_{i}^{m}$, which is peculiar to the mission in which the *i^th^* drone subsystem is involved at the time of the BIT. This gives us the general state of efficiency $F_{i}$ according to the mission *m-type*:

(20) $$F_{i}=\frac{f_{i}^{s}}{f_{i}^{m}}$$

For example, in a mission of very short duration, the state of the batteries $f_{BatteryPack}^{s}$ is acceptable even if partially degraded to 50% because it is called to respond only with a fraction of the stored energy; on the contrary, if the mission requires the identification of crude oil leaks, the sensor that detects the hydrocarbons is required for maximum efficiency.

In our case, *fuzzification* consists of the conversion of input values (which, in our case, is degradation value) into linguistic values with a degree of membership that can be given, as in our case, by continuous functions peculiar to each single subsystem. As far as control is concerned, we set each defined “if then” rule, and the degree of applicability of the same is calculated from the minimum between the two membership values of the variables [20].

The defuzzification depends on the conversion of fuzzy function values into clear values; this is organized through the control rule, which, in our case, can be easily schematized as follows:

(21) $$z=\frac{\sum_{i=1}^{j}F_{i}{}^{*}\cdot z_{i}}{\sum_{i=1}^{j}z_{i}}$$

where $z_{i}$ is the degree of applicability of the rule, $F_{i}{}^{*}$ is the central value of the *membership function*, and z can be considered as the overall degradation variable: Once the acceptability limit has been set, $z_{min}$, the drone will go off-line if the following results:

(22) $$IF\;z<z_{min}\;THEN\;Drone\;GO\;Off-Line$$

The minimum is posed as:

(23) $$z_{min}=\;\frac{\partial R_{k/n}}{\partial t}=94.58\%$$

Thus, the $z_{min}$ is completely defined [21].

### 2.5. Vehicle Motion

Each individual vehicle is programmed to proceed to the vectorial sum of two speeds: The first is purely random, the second is influenced by the proximity of other drones, and has the purpose of removing the vehicle from the others in order to not “pile them up” with the others. The final purpose is precisely that of spreading the swarm uniformly at a constant density within a closed volume: In our case, we refer to a surface because the bathymetric variations are very modest compared to the displacement on the horizontal plane.

The vehicle proceeds with two speed components, the first is ${\vec{v}}_{random}$: It is a pure casual speed/direction combination. The full expression is:

(24) v→random(t)=[vrandomx(t)vrandomy(t)vrandomz(t)]

Every direction comprises two components

vrandom(t−Δt) is the speed that takes into account the “history” of the vehicle

$R_{}\left(\Delta t\right)$ is a random zero mean vector

$\delta_{}\left(\Delta t\right)$ is an algebraic scalar that modifies the direction

Therefore, the full expression becomes:

(25) v→random(t)=[vrandomx(t−Δt)+Rx(Δt)·δx(Δt)vrandomy(t−Δt)+Ry(Δt)·δy(Δt)vrandomz(t−ऴt)+Rz(Δt)·δz(Δt)]

However, we impose than the AUV moves constantly at a defined depth, a sort of bi-dimensional “moving plane”; therefore:

(26) $$\delta_{z}\left(t\right)\approx 0$$

Due to the negligible movement on the vertical plane, we have:

(27) v→random(t)=[vrandomx(t−Δt)+Rx(Δt)·δx(Δt)vrandomy(t−Δt)+Ry(Δt)·δy(Δt)vnz(t−Δt)]

The speed of the single vehicle is influenced by the position of the other vehicles: We define ${\vec{v}}_{swarm}\left(t\right)$ as the second component of speed that “diffuses” the swarm in the full area as:

(28) $${\vec{v}}_{swarm}\left(t\right)=\{\begin{array}{c} \overset{n}{\underset{i=1}{\sum }}\frac{{\left|r-r_{i}\right|}^{3}}{\left|r-r_{i}\right|}\\ \left|r-r_{i}\right|\leq d_{min} \end{array}$$

where

n is the full number of drones

r the distance of the considered drone

$r_{i}$ the distance of the ith drone

$d_{min}$ the min *collision* distance of the drone

We mean with “*minimum collision distance*” the minimum distance that, at maximum speed, allows enough time for two drones on a collision course to exchange position and speed data and to elaborate and implement an escape route. Depending on the optical transducer chain, this was set at 50 m.

Thus, the total speed of the single vehicle is:

(29) $${\vec{v}}_{TOT}\left(t\right)={\vec{v}}_{random}\left(t\right)+{\vec{v}}_{swarm}\left(t\right)$$

Thus, the complete expression is:

(30) v→TOT(t)={[vrandomx(t−Δt)+Rx(Δt)·δx(Δt)vrandomy(t−Δt)+Ry(Δt)·δy(Δt)viz(t−Δt)]+[∑i=1n|rx−rix|3|rx−rix|∑i=1n|ry−riy|3|ry−riy|0]|r−ri|≤dmin

It is interesting to note that, if the drones were released in a single point and authorized to move in a closed and defined area, behaving in the substance as gas particles that expand, the ${\vec{v}}_{swarm}\left(t\right)$ is transitory only. In fact, this speed increases in the first period, as soon as the drones start; then, once “expanded” in the whole volume, and the “center of gravity” of the swarm being stabilized, its value is zero.

**Figure 5:**
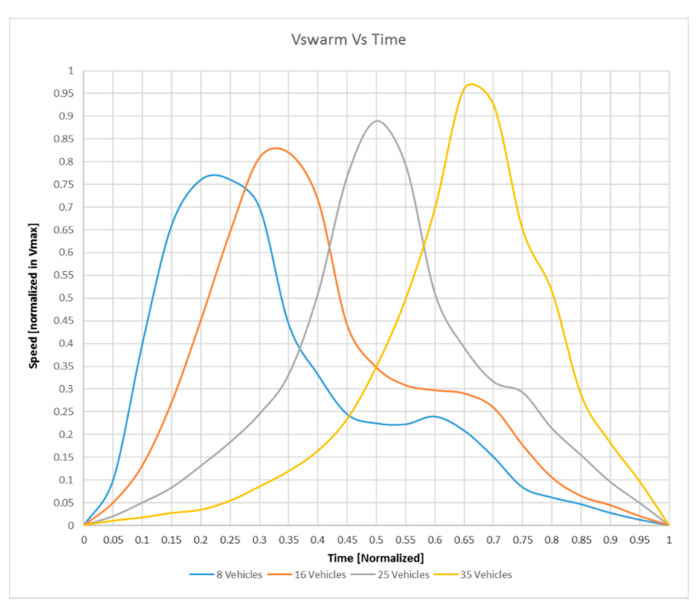
Velocity of the swarm vs. time: Parametric curves with 8, 16, 25, and 35 vehicles (time and speed are normalized).

In Figure 5, it is possible to see the evolution of the ${\vec{v}}_{swarm}\left(t\right)$ as a function of time. The time axis is normalized to 1 to be able to compare trends while the speeds are also normalized to 1, which indicates the maximum speed of the single drone. It is noted that as the number of swarm members increases, swarm speeds are always higher but are reached later. All curves tend to zero speed as soon as the whole area is saturated with drones.

### 2.6. Swarm Diffusion

Now, we can model our system according to the Fokker–Planck equation: It describes the temporal evolution of the density of the probability function of a particle position and can be generalized to other observable entities. From the macroscopic point of view, we can consider the drone as a dispersed particle over a square area: Its movement is random within the closed area, very similar to Brownian motion (the differences in the vertical plane are very modest), and, if it gets too close to another, it receives a repulsion similar to an elastic shock.

All this is expressed with the time evolution of the probability density:

(31) $$\frac{\partial p\left({\mathbb{P}}_{xy},t\right)}{\partial t}=-\nabla \left[{𝕗}_{1}\left({\mathbb{P}}_{xy},t\right)\cdot p\left({\mathbb{P}}_{xy},t\right)\right]+\frac{1}{2}\left|d_{min}\right|\cdot \nabla^{2}\left[{\left({𝕗}_{2}\left({\mathbb{P}}_{xy},t\right)\right)}^{2}\cdot p\left({\mathbb{P}}_{xy},t\right)\right]$$

where:

${\mathbb{P}}_{xy}$ is the position of the vehicle in the horizontal *(x,y)* plane;

p is the density of the swarm at time *t*; 

$\nabla p\left({\mathbb{P}}_{xy},t\right)d{\mathbb{P}}_{x}d{\mathbb{P}}_{y}$ is the probability to find a UAV position ${\mathbb{P}}_{xy}$ in the area defined by $d{\mathbb{P}}_{x}$ and $d{\mathbb{P}}_{y}$ at time *t*;

${𝕗}_{1}\left({\mathbb{P}}_{xy},t\right)$ is a direction and describes the deterministic motion based on information provided by the environment and the information indirectly provided by other UAVs via the environment;

${𝕗}_{2}\left({\mathbb{P}}_{xy},t\right)$ describes the random component of the motion;

For ${𝕗}_{1}\left({\mathbb{P}}_{xy},t\right)$, we consider the definition:

(32) $${𝕗}_{1}\left({\mathbb{P}}_{xy},t\right)=\sqrt{1-\mu \left(p\right)}$$

where:

(33) $$\mu \left(p\right)=\frac{min\left(p,\varphi_{r}\right)}{\varphi_{r}}$$

in which $\varphi_{r}$ is the maximum expected density of a UAV in the area defined by ${\mathbb{P}}_{x}$ and ${\mathbb{P}}_{y}$.

For ${𝕗}_{2}\left({\mathbb{P}}_{xy},t\right)$, we consider the definition:

(34) $${𝕗}_{2}\left({\mathbb{P}}_{xy},t\right)=\varphi_{a1}+\varphi_{a2}\cdot \mu {\left(p\right)}^{\varphi_{a3}}$$

$\varphi_{a1}$ is the random factor;

$\varphi_{a2}$ and $\varphi_{a3}$ are the factors due to the virtual collision and the proximity of the other vehicles. 

Now, consider a random rectangular area of the surface, of the type:

(35) $$D=\left[x_{a},x_{b}\right]\times \left[y_{a},y_{b}\right]$$

Of this area, we are going to consider the probability density at time $t_{\gamma }$ by integrating Equation (31):

(36) $$\{\begin{array}{c} {\mathbb{P}}_{xy}=\iint_{D}^{}-\nabla \left[{𝕗}_{1}\left({\mathbb{P}}_{xy},t\right)\cdot p\left({\mathbb{P}}_{xy},t\right)\right]+\frac{\left|d_{min}\right|}{2}\cdot \nabla^{2}\left[{\left({𝕗}_{2}\left({\mathbb{P}}_{xy},t\right)\right)}^{2}\cdot p\left({\mathbb{P}}_{xy},t\right)\right]dxdy\\ t=t_{\gamma } \end{array}$$

Therefore, we have:

(37) $${\mathbb{P}}_{xy}=\iint_{D}^{}-\nabla \left[{𝕗}_{1}\left({\mathbb{P}}_{xy},t\right)\cdot p\left({\mathbb{P}}_{xy},t\right)\right]+\frac{\left|d_{min}\right|}{2}\cdot \nabla^{2}\left[{\left({𝕗}_{2}\left({\mathbb{P}}_{xy},t\right)\right)}^{2}\cdot p\left({\mathbb{P}}_{xy},t\right)\right]dxdy$$

and

(38) $${\mathbb{P}}_{xy}=\overset{y_{b}}{\underset{y_{a}}{\int }}\left(\overset{x_{b}}{\underset{x_{a}}{\int }}-\nabla \left[{𝕗}_{1}\left({\mathbb{P}}_{xy},t\right)\cdot p\left({\mathbb{P}}_{xy},t\right)\right]+\frac{\left|d_{min}\right|}{2}\cdot \nabla^{2}\left[{\left({𝕗}_{2}\left({\mathbb{P}}_{xy},t\right)\right)}^{2}\cdot p\left({\mathbb{P}}_{xy},t\right)\right]dx\right)dy$$

**Figure 6:**
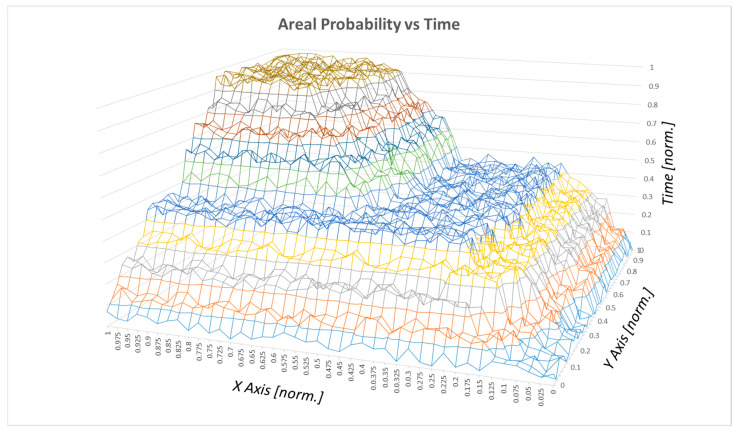
Surface probability vs. time.

The diffusive parameters of Equation (34) are presented in Table 2:

The probability correlated to the surface (area) is shown in Figure 6:

### 2.7. Simulation

We have considered that the swarm must be composed of 25 UAVs because they were uniformly distributed over the entire surface. Obviously, in operating conditions in a wider scenario, we can imagine the presence of nine drones in stand-by conditions that would occur in the case of the malfunction of one or more elements of the swarm.

In our simulation, at time *t* = 0, all vehicles are put into the sea all at the same time in the *“Start point 0,0”*, which corresponds to the corner of the square area. This is a real case: As our drones do not need a support boat, they can be released into the sea like a simple dinghy from a slide.

The diffusive parameters of Equation (25) are the same as those in Table 2:

The simulation was performed with the combined Matlab^®^/Simulink^®^ tool: The results were validated separately on a sample of 30 specific points. The values were derived from experimental tests.

**Figure 7:**
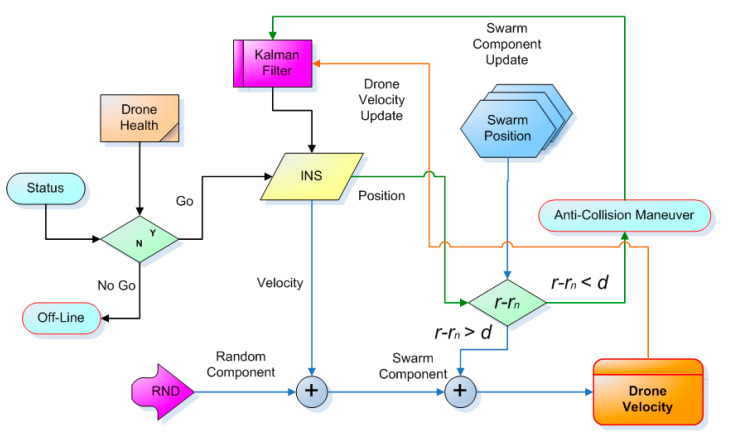
Block diagram of the essential functions of a single drone and the interaction with the environment.

It starts (see Figure 7) from the previous *status* of the drone: its *health* (evaluated in accordance with the self-purging redundancy paradigm) is then inserted if this is off-line; otherwise, it is declared operational. In this case, it triggers the navigation cycle based on the inertial system (INS), which evaluates expression (30) of Section 2.6, which provides the speed to which a random part is first added and then the swarm component is added. First, however, it is also assessed on the basis of the proximity of the other drones: If one of them is too close, an automatic anti-collision procedure is started, which will then update the Kalman filter register. Eventually, we reach the speed of the drone: This data will also update the Kalman filter register. At the end, the speed data will return to the inertial computer (INS), which can be interrupted at any time by a possible malfunction that will make the health condition critical.

**Figure 8:**
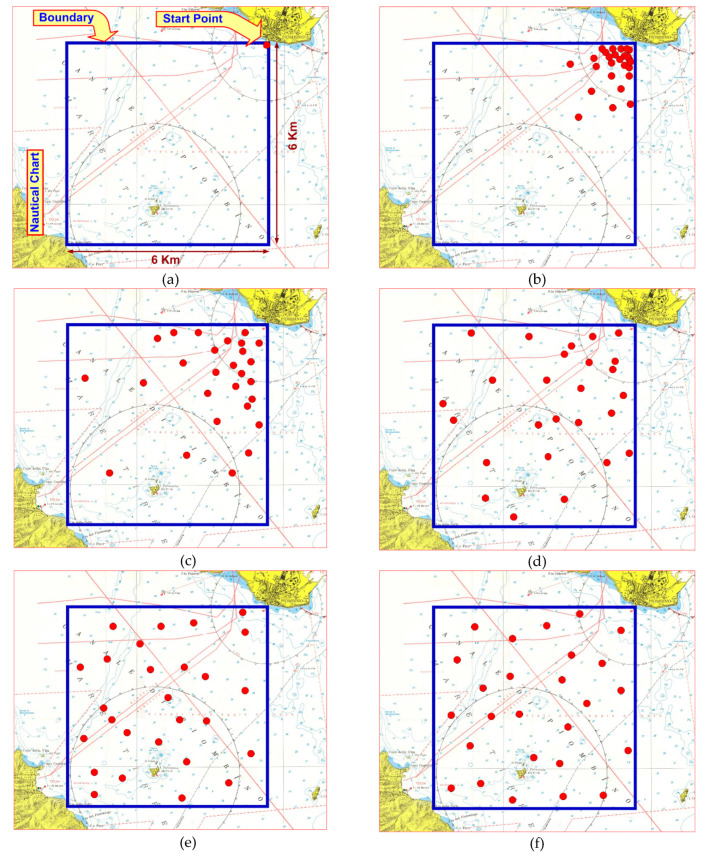
“Snapshots” over time, at intervals of 0 (**a**), 0.5 (**b**), 1 (**c**), 4 (**d**), 8 (**e**), and 12 h (**f**).

In Figure 8, we show how the probability density varies spatially by taking three “snapshots” over time, at intervals of 0, 0.5, 1, 4, 8, and 12 h. 

In the initial conditions, *t* = 0, all 25 drones are entered at point “*0.0*”: The point chosen is precisely the port of Piombino. Once slipped into the sea, vehicles are free to spread throughout the previously defined area. 

When a drone reaches the limit of the surveillance area, it behaves exactly like a gas particle: The AUV “bounces” on the limit with a purely elastic impact, that is, it changes its velocity vector by a complementary angle at 180°, maintaining its absolute value in speed.

As mentioned above, the variations in the depth plane are absolutely modest compared to the displacements on the horizontal plane: This is due to the fact that the drone was designed for use in shallow waters and the payload, in our case study, is designed for exclusive surface use.

We observe the progressing development of the simulation. Figure 8a illustrates the blue square in which the simulation superimposed on the nautical chart of the Piombino strait takes place: All the drones (red dots) are inserted in the *start point* corresponding, for accuracy, to the commercial port of *Salivoli*. At time *t* = 0, they are all very close, almost coincident. In Figure 8b, we see the expansion of the drones at *t* = 0.5 h.

They are still concentrated in the corner but begin to push toward the center of the square. In Figure 8c,d, we still see two other phases of the expansion in which the drones begin to frequent even the most distant areas. In Figure 8e, we have a very low “swarm speed”; moreover, the probability density is almost constant. In Figure 8f, the evolution is completed: From a dynamic point of view, the drones move but their density is constant and the *swarm speed* is zero.

## 3. Conclusions

By means of simulations, we expose the feasibility of a swarm of low-cost, low-intelligence drones that can act as a sentinel for a stretch of sea. First of all, we introduce the vehicles with a low and uncoordinated intelligence that have the ability to be resilient to the loss of one or more members thanks to the raising of the overall reliability based on the reasonable elevation of the number of members.

The paradigm we have investigated is based on a series of constraints that are very loose between the various members of the swarm: The autonomy of each individual is maximum while the interaction with neighbors is limited to proximity; from here, the particle model is developed.

Then, we study the swarm behavior for UAVs, which is formalized with a microscopic diffusion model in order to justify the simple single-drone program.

In the final part, we show that the drones are not a path defined by a computer center that coordinates the movements and, therefore, proceeds in a completely random way with the only constraint to move away from each other. Once this process has started, they tend to become more and more uniformly distributed on the surface of the assigned sea until they reach a constant probability over the entire surface, that is, after a time of 12 h.

This means that the swarm, despite moving in a completely random way, manages to “fill” the entire predetermined area in a short time. The simulation defines and shows the behavior of a swarm of underwater drones after their insertion in a closed section of the sea, from a single point and their course for the whole area assigned to them. This will help us to define the characteristics of the drones (and, above all, their guidance and control subsystems) that we are developing.

## Figures and Tables

**Table 1:** AUV: General characteristics and performances (estimated).

Displacement	Length	Beam ^1^	Wing Span	Cruise Speed		Range ^2^	Endurance ^2^	Depth
59 kg	0.920 m	0.22 m	0.445 m	18 kn	34 km/h	~5700 km	168 h	200 m

^1^ Without annular wing. ^2^ At cruise speed.

**Table 2:** Parameters of the simulation.

Parameter	Value
# of Vehicles	25
$\varphi_{r}$	0.0055
$\varphi_{a1}$	0.81
$\varphi_{a2}$	3
$\varphi_{a3}$	0.099
